# A Modified-Delphi Consensus on the Management of Patients with FLT3-Mutated AML

**DOI:** 10.3390/cancers18050770

**Published:** 2026-02-27

**Authors:** Jacopo Olivieri, Emanuele Angelucci, Roberto Cairoli, Maria Paola Martelli, Massimo Martino, Cristina Papayannidis, Simona Sica, Maria Teresa Voso, Adriano Venditti

**Affiliations:** 1 Hematology Clinic and Cellular Therapy Center, Azienda Sanitaria Universitaria Friuli Centrale (ASUFC), 33100 Udine, Italy; jacopo.olivieri@asufc.sanita.fvg.it; 2 Hematology and Cellular Therapy Unit, IRCCS San Martino Hospital, 16132 Genova, Italy; emanuele.angelucci@hsanmartino.it; 3 Department of Hematology, Oncology and Molecular Medicine, Niguarda Cancer Center, ASST Grande Ospedale Metropolitano Niguarda, 20161 Milan, Italy; roberto.cairoli@ospedaleniguarda.it; 4 Hematology and Clinical Immunology Section, Center for Hemato-Oncology Research, Department of Medicine and Surgery, University of Perugia, 06156 Perugia, Italy; maria.martelli@unipg.it; 5 Hematology Division, ‘Santa Maria della Misericordia’ Hospital, Azienda Ospedaliera di Perugia, 06156 Perugia, Italy; 6 Hematology and Stem Cell Transplantation and Cellular Therapies Unit (CTMO), Grande Ospedale Metropolitano Bianchi-Melacrino-Morelli, 89133 Reggio Calabria, Italy; massimo.martino@ospedalerc.it; 7 Hematology Institute Seràgnoli, IRCCS Azienda Ospedaliero-Universitaria di Bologna, 40100 Bologna, Italy; cristina.papayannidis@unibo.it; 8 Section of Hematology, Department of Radiological and Hematological Sciences, Catholic University, 20123 Rome, Italy; simona.sica@unicatt.it; 9 Department of Laboratory and Hematological Sciences, Fondazione Policlinico Gemelli IRCCS, 00168 Rome, Italy; 10 Onco-Hematology Advanced Diagnostics UOSD, Policlinico Universitario Tor Vergata, 00133 Rome, Italy; voso@med.uniroma2.it; 11 Onco-Hematology Department, Fondazione Policlinico Tor Vergata, 00133 Rome, Italy

**Keywords:** acute myeloid leukemia, FLT3 inhibitor, consensus

## Abstract

**Background/Objectives**: The emergence of FLT3 inhibitors (FLT3i) has radically transformed the prognostic and therapeutic landscape for FLT3-mutated Acute Myeloid Leukemia, stimulating the need for comprehensive and structured clinical guidance. **Methods**: We aimed to develop evidence-based recommendations spanning the entire disease continuum of FLT3-mutated AML from leading Italian experts through a modified Delphi consensus process. **Results**: The panel achieved a high degree of agreement on specific interventions covering diagnostic testing, upfront FLT3i integration, role of allogeneic hematopoietic cell transplantation (allo-HSCT), Minimal Residual Disease (MRD) monitoring, and relapsed/refractory (R/R) strategies. Key recommendations mandate that analysis for both FLT3-ITD and FLT3-TKD mutations is required at diagnosis, with capillary electrophoresis or NGS as preferred methods. All fit patients with FLT3m-AML must receive intensive chemotherapy plus a FLT3i (midostaurin or quizartinib) and be evaluated for allo-HSCT. For unfit patients, the current standard of HMA + venetoclax is considered suboptimal, making the search for alternative strategies imperative. MRD monitoring using available molecular or flow cytometry markers is recommended to assess relapse risk and to optimize the allo-HSCT strategy. In the R/R setting, retesting the FLT3 status is mandatory, and gilteritinib is the standard treatment, serving as a bridge-to-transplant and for post-HSCT maintenance. **Conclusions**: The integration of FLT3i has shifted FLT3m-AML into a more favorable intermediate prognostic category, enhancing the role of curative strategies like allo-HSCT. This consensus paper provides a structured evidence-based comprehensive guide, translating complex data into clear actionable clinical recommendations that minimize practice variability and ultimately optimize management for this high-risk population.

## Simple Summary

Acute myeloid leukemia (AML) is a fast-growing blood cancer. Many patients have alterations in a gene called FLT3, which were once linked to poor outcomes. New targeted medicines called FLT3 inhibitors have greatly improved treatment, but their use requires clear guidance. A group of Italian experts reviewed the available evidence and agreed on practical recommendations for treating patients with AML with FLT3 alterations. They recommend that all patients are tested for FLT3 alterations when they acknowledge to be sick. Patients who can receive intensive therapy should receive standard chemotherapy combined with an FLT3 inhibitor and be assessed for a potentially curative stem cell transplant. For patients who cannot receive intensive treatment, current options are often not effective enough, and better therapies are urgently needed. Monitoring small amounts of remaining leukemia after treatment is important to estimate relapse risk and guide transplant decisions. If the disease returns, FLT3 testing should be repeated, and treatment with the FLT3 inhibitor gilteritinib is recommended. Overall, FLT3 inhibitors have improved the outlook for patients with FLT3-mutated AML. This consensus provides clear, evidence-based advice to help doctors deliver more consistent and effective care.

## 1. Introduction

Mutations in the FMS-like tyrosine kinase 3 (FLT3) gene represent one of the most frequent and clinically significant abnormalities of acute myeloid leukemia (AML), affecting approximately 30% of patients. FLT3 mutations comprise FLT3-internal tandem duplication (ITD) and FLT3-tyrosine kinase domain point mutations (mainly D835) [1,2] that imply relevant differences in clinical presentation, prognosis and management of AML. FLT3-ITD mutations are found in approximately 20–25% of cases and frequently co-occur with other mutations, such as DNMT3A and NPM1 [2,3]. Clinically FLT3-ITD mutated AML is characterized by higher leukocyte counts, an increased risk of relapse, and historically with a shorter overall survival compared to patients with wild-type FLT3. This adverse prognostic profile has led to the classification of FLT3-ITD mutations with a high allelic burden and absence of NPM1 mutation as a high-risk feature in the 2017 European LeukemiaNet (ELN) AML risk stratification system. By contrast, FLT3-tyrosine kinase domain (TKD) mutations are less frequent, occurring in about 5–10% of AML patients and their prognostic significance is controversial [4,5,6].

The introduction of FLT3 kinase inhibitors has significantly reshaped the management of AML harboring FLT3-ITD and FLT3-TKD mutations. FLT3 inhibitors (FLT3i) include both first-generation multi-kinase inhibitors (sorafenib, midostaurin), and more potent, selective second-generation inhibitors (gilteritinib, quizartinib). Moreover, FLT3i can be distinguished by their activity on different FLT3 mutations: type I (gilteritinib, midostaurin) target both FLT3-ITD and FLT3-TKD, whereas type II (quizartinib, sorafenib) only inhibit FLT3-ITD. The integration of these targeted agents into standard chemotherapy regimens has significantly improved outcomes for patients with FLT3-mutated AML, in the frontline, and more recently in the relapsed/refractory setting [7,8]. These therapeutic advantages have led to a change in the prognostic classification of FLT3-mutated AML, from an unfavorable category to an intermediate category according to the ELN 2022 classification [4,9]. Ameliorated response rates have also increased the possibility to access to allogeneic hematopoietic stem cell transplantation (allo-HSCT), allowing more patients to be cured [10,11].

Also, in the setting of relapsed or refractory (R/R) FLT3-mutated AML the development of selective FLT3i has been a significant breakthrough. The phase III ADMIRAL trial demonstrated a statistically significant survival benefit for gilteritinib monotherapy as compared to salvage chemotherapy in R/R patients with FLT3 mutations, establishing gilteritinib as a standard of care in R/R FLT3-mutated AML [12]. Another key study, the Phase III QuANTUM-R trial, showed a survival advantage for the selective FLT3i quizartinib over salvage chemotherapy in a similar patient population [13]. Ongoing research is now focused on combining FLT3i with other targeted agents, such as the BCL-2 inhibitor venetoclax, to overcome resistance and achieve deeper, more durable remissions. Clinical trials evaluating these combination regimens are a major focus of current research, with promising early results showing high response rates, even in patients who have been previously exposed to FLT3i [7].

This rapid change in the prognostic and therapeutic landscape of FLT3-mutated AML highlights the need for a comprehensive reappraisal of the management strategy of this disease to optimize patient outcomes. As clinical evidence continues to evolve, a consensus on best practices is essential to guide therapeutic decision-making in this high-risk patient population.

The aim of this consensus Delphi is to provide a guide for the management of patients with FLT3-mutated AML from the first to subsequent lines of treatment from an Italian panel of experts.

## 2. Materials and Methods

### 2.1. Selection of Steering Committee and/or Panelists

The steering committee (SC) was comprised of three Italian hematologists, of whom one was an expert in the consensus methodology. Every member of the steering committee had prior experience drafting consensus agreements and national and international guidelines. The steering committee identified six panelists based on their qualifications and track record in the clinical management of AML and invited them to participate. A restricted number of panelists was chosen to maximize the interaction and the discussion during the project.

### 2.2. Literature Search

To inform the development of the consensus statements, a standardized literature search on recent clinical trial data, current guidelines, and key publications on FLT3-mutated AML management was conducted by the SC. The methodology was standardized to ensure that all core concepts were covered but did not follow a formal systematic review protocol. MEDLINE was searched through PubMed using a combination of the following keywords as MeSH terms or free words for variations and emerging terminology on the following concepts: acute myeloid leukemia, FLT3 mutation, study/trial, frontline or relapsed/refractory setting, FLT3 inhibitor. Details are provided in Appendix A, Appendix B, Appendix C. The search was limited to human studies and focused on English-language articles published within the last ten years (2015–2025), with priority given to randomized controlled trials (RCTs), meta-analyses, and major society guidelines. The literature review was integrated by suggestions of the panelists.

### 2.3. Preparatory Research and Consensus Process

Statements were drafted based on the synthesized evidence, and a modified Delphi procedure was employed to validate them, reported according to the ACCORD guidelines [14]. A two-round Delphi was initially planned to allow for iteration. Panelists received the statements by e-mail and were asked to rate each on a 5-point Likert scale: “1 = Strongly Disagree”, “2 = Disagree”, “3 = Neither agree nor disagree”, “4 = Agree”, and “5 = Strongly agree”. The threshold to define consensus was defined a priori as ≥80% of participants voting “Agree” or “Strongly agree.” Panelists were also able to provide anonymous free-text comments to justify their vote or suggest modifications to the wording. After each round, comments were reviewed by J.O. and V.A., and semantic refinements were made to improve clarity. Between rounds, participants received anonymized feedback, including aggregated voting results and relevant comments, to facilitate reflection and alignment.

### 2.4. Participation and Processes

The Delphi panel included a total of eight experts, of whom two were members of the steering committee (V.A. and C.R.). After the first round of voting, held in April 2025, all statements achieved a consensus; the steering committee evaluated the responses and free-text comments written by the panelists and deemed it unnecessary to hold a second round of voting. J.O. elaborated the responses in May 2025. The steering committee and the panelists met online in June 2025 and discussed finding an agreement on the final semantic proposal of the statements. An in-person meeting was convened in September 2025 to finalize the recommendations and to critically review and summarize their supporting evidence.

## 3. Results

The first round of voting demonstrated a high level of agreement across all statements. Based on these results, the SC determined that a second round was unnecessary. However, full agreement was not reached for most statements, and several comments or corrections were anonymously posted by panelists. Hence, during the in-person meeting held in Rome in September 2025, the statements were further discussed and refined to enhance clarity and ensure a more focused message. The list of statements initially voted, and a summary of most relevant discussions are available as Appendix C.

### 3.1. Diagnosis, Risk Stratification, and First Therapeutic Decision

Statement: “FLT3-ITD mutation analysis is recommended at diagnosis in all AML patients eligible for antileukemic treatment. Capillary electrophoresis is the detection method of choice. Next Generation Sequencing (NGS) is an alternative option, being equally informative when positive”.

First round: 8 out of 8 (100%) panelists chose “Strongly agree”.

This recommendation was adapted from Italian guidelines on “Advanced diagnosis of AML” by the Italian Society of Experimental Hematology (SIES) published on the National Guideline System (Sistema Nazionale Linee Guida). The recommendation was made upon the following evidence.

The detection of FLT3-ITD mutation at diagnosis is crucial in AML management, as its presence significantly impacts prognosis and contributes to risk stratification according to ELN 2022 and ELN 2024 [9,15]. Capillary electrophoresis is the preferred method for FLT3-ITD testing due to its high sensitivity in detecting ITD size and allelic burden: additional peaks in exons 14/15 indicate duplications, and the allelic ratio is calculated as the ratio mutant/wild-type peak areas. Use of NGS has been recently advocated to substitute for traditional capillary electrophoresis. The SIES guideline reviewed the literature on the diagnostic accuracy of targeted NGS and traditional capillary electrophoresis, demonstrating a high correlation between the two methods and high sensitivity of NGS [16]. However, also in line with ELN 2022, the SIES guideline recognizes that NGS may be unreliable for accurately determining the presence of FLT3-ITD due to their heterogeneity in size, as longer FLT3-ITDs may be missed [17,18], although it may provide some information on the presence of alternative FLT3 mutations. In addition, standard NGS may fail to detect microclones with an allelic ratio (AR) below 0.05; therefore, capillary electrophoresis should be performed in all cases that test negative by NGS. Higher sensitivity NGS technologies may overcome this issue [19,20]. On the other hand, since turnaround time for NGS is usually prolonged for technical reasons, while that for traditional capillary electrophoresis for FLT3-ITD is generally <7 days, NGS cannot yet be recommended as standard procedure for FLT3-ITD mutation testing. These figures may change soon, with the ongoing improvements in targeted NGS technology.

Statement: “FLT3-TKD mutation analysis is recommended at diagnosis in all AML patients eligible for antileukemic treatment. Capillary electrophoresis is the detection method of choice. NGS is an alternative option, being equally informative when positive”.

First round: 8 out of 8 (100%) panelists chose “Strongly agree”.

In contrast to FLT3-ITD, FLT3-TKD does not consistently correlate with worse outcomes in large cohorts: no significant survival impact was observed in meta-analyses [21], while some studies suggested a favorable prognosis in patients with coexisting NPM1 mutation [4,22]. Based on the above data, the ELN 2022 guidelines did not include FLT3-TKD as a prognostic marker, distinct from FLT3-ITD [5]. Despite its neutral prognostic role, FLT3-TKD testing is recommended to guide frontline and relapse therapy selection, as effective FLT3i targeting FLT3-TKD is currently available [11,23].

Statement: “All patients with FLT3-mutated AML who are fit for intensive chemotherapy must receive intensive chemotherapy plus a FLT3i regardless of other co-occurring mutations”.

First round: 7 out of 8 (87.5%) panelists chose “Strongly agree” and one panelist (12.5%) chose “Agree”.

The current standard of care for newly diagnosed, fit patients with FLT3-ITD-mutated AML is the combination of intensive chemotherapy with a FLT3i. This practice is strongly supported by the phase III randomized pivotal RATIFY trial, which established the superiority of adding midostaurin to standard 7 + 3 chemotherapy in newly diagnosed FLT3-mutated AML (both ITD and TKD) [11]. The trial (*n* = 717) showed a 51.4% 4-year overall survival (OS) rate in the midostaurin arm compared to 44.3% with chemotherapy alone (HR 0.78, *p* = 0.009). Although the complete remission (CR) rate was not improved (59% vs. 54%, *p* = 0.15), the significant survival advantage was driven by reduced relapse risk, particularly in patients with a high FLT3-ITD allelic ratio (≥0.7), who benefited most (HR 0.66) [11]. Toxicity was manageable, without a significant increase in grade ≥3 adverse events. Updated survival results with a 10-year follow-up confirmed durable event-free survival benefit for midostaurin arm [24]. The QUANTUM-First trial repeated a similar trial design by including the addition of a second generation FLT3i to IC in 539 FLT3-ITD-mutated AML patients. The primary endpoint OS was statistically improved with quizartinib vs. placebo (median: 31.9 vs. 15.1 months; HR 0.78, *p* = 0.032). Some potential cardiac AE specific to quizartinib emerged as relevant concerns: QTc prolongation was more common than placebo (13.6% vs. 4.1%) and, although rare, serious and fatal AE from ventricular arrhythmia were reported with quizartinib [25].

More recently, a phase Ib trial evaluated gilteritinib combined with induction chemotherapy in untreated FLT3-mutated AML (both ITD and TKD) for 36 evaluable participants, the composite CR (CRc) was 89% (conventional CR were 83%) and the median OS was 46.1 months; gilteritinib was well-tolerated across all tested doses (40–200 mg) and with either of the two chemotherapy backbones (7 + 3 with idarubicin or daunorubicin) [26]. Another phase randomized II trial compared gilteritinib versus midostaurin in combination with intensive chemotherapy (7 + 3 with daunorubicin 90 mg/m^2^) in 177 newly diagnosed AML patients with FLT3 mutations (both TKD and ITD). Overall, 85.6% of patients in the gilteritinib arm achieved CRc (CR 40%) compared to 72.4% (CR 47.1%) with midostaurin (*p* = 0.042), while the rate of minimal residual disease (MRD) negativity assessed using flow cytometry was not significantly different [27]. The ongoing phase 3 randomized HOVON-156 trial is testing in a similar setting gilteritinib versus midostaurin in combination with intensive chemotherapy (induction with 7 + 3 with daunorubicin 60 mg/m^2^), also including one-year maintenance with the FLT3i [28].

Real life data on the use of midostaurin in combination with IC were comparable to that reported in the pivotal clinical trial in a recent GIMEMA survey [29].

Co-mutations in TP53, NRAS or other myelodysplasia-related genes may affect the prognosis, but little evidence is available on their effect on FLT3 inhibition. While co-occurring mutations influence AML biology and can modify the risk category according to ELN 2022, current evidence suggests that they should not alter the initial therapeutic approach in FLT3-ITD AML. A large study by Jahn et al. [3] analyzed the mutational landscape and its clinical significance in 475 patients with FLT3-mutated AML enrolled on the pivotal CALGB 10603/RATIFY trial and showed that OS did not significantly change in patients treated with midostaurin and having co-mutations in DNMT3A (39%), WT1 (21%), TET2 (12%), NRAS (11%), RUNX1 (11%), PTPN11 (10%), and ASXL1 (8%); TP53 mutations were rare (*n* = 4; 0.8%), precluding definitive conclusions. Moreover, in a retrospective exploratory study involving 318 of 549 patients with FLT3-ITD mutated AML from the RATIFY trial, treatment with midostaurin was associated with improved outcomes in all three risk subgroups, according to 2017 ELN classification [6]. In the few cases with inv (16), the addition of a FLT3i (TKi) to intensive chemotherapy resulted in a gradual reduction in the pathological transcript during the maintenance phase, which consisted of FLT3i monotherapy, ultimately reaching undetectable levels [30]. Therefore, based on current knowledge, the presence of other genetic abnormalities should not modify the therapeutic choice on the use of a FLT3i nor affect the decision-making process in patients with FLT3-mutated AML.

Statement: “All patients with FLT3-ITD AML who are fit for intensive chemotherapy should be evaluated for allogeneic transplant eligibility”.

First round: 7 out of 8 (87.5%) panelists chose “Strongly agree” and one panelist (12.5%) chose “Agree”.

The ELN 2022 guidelines provide a standardized framework for risk stratification in AML, including cases with FLT3 mutations. In contrast to prior classifications that incorporated allelic ratio (FLT3-ITD/FLT3-wild-type) and NPM1 co-mutation status, the updated ELN criteria uniformly categorize FLT3-ITD mutations (without adverse-risk genetic lesions) as intermediate-risk, irrespective of allelic burden or concurrent NPM1 mutations [4]. This adjustment reflects evidence that even the low-allelic-ratio FLT3-ITD retains an adverse prognostic relevance [31] and FLT3i (e.g., midostaurin, gilteritinib) improve outcomes across subgroups [11,12].

Allo-HSCT in first complete remission (CR1) remains a cornerstone of therapy for FLT3-ITD AML. Several studies underline the synergy towards a better outcome with an induction strategy comprising a FLT3i followed by allo-HSCT [32]. Firstly, the addition of a FLT3i to IC may increase the access to allo-HSCT (after CR1: for RATIFY trial: 28.1% in the midostaurin arm vs. 22.7% in the placebo group; for QUANTUM-First trial: 31.3% with quizartinib vs. 26.9% with placebo) [10,11]. Secondly, among those who received allo-HSCT in CR1, OS tend to be better in patients randomized to the arm including the FLT3i. The synergistic effect of FLT3i and allo-HSCT was demonstrated in a post-hoc analysis of the QUANTUM-First trial, where quizartinib treatment and allo-HSCT in CR1 were an independent predictor of better OS in multivariable analyses, both showing an estimated reduction in death risk of 40% or greater [10]. Similar added benefits of allo-HSCT to intensive induction with FLT3i are reported in a post-hoc analyses of the RATIFY trial [3,11] and from retrospective real-world studies [33,34].

Overall, the decision whether to proceed with transplantation in patients with FLT3-ITD AML, similarly to all other patients with AML, should be based on a risk-adapted strategy, considering the difference in outcome between the risk of relapse/death without transplantation and non-relapse mortality [31].

Therefore, the panel suggests that, in situations where non-relapse mortality (NRM) is estimated to be very high (greater than 35% with scores currently in use such as HSCT-CI) and the risk category is intermediate, allogeneic transplantation is not indicated outside of clinical trials. Similarly, as discussed later in this paper, when the risk of relapse is very low, as FLT3-ITD AML with NPM1 co-mutation, and MRD negative after induction, the allo-HSCT could be omitted [35].

Statement: “Current standard treatment for patients with FLT3-ITD AML who are unfit for intensive chemotherapy is represented by the combination of a hypomethylating agent and venetoclax, which, however, should be considered suboptimal in this subset of AML. Alternative options include enrollment in clinical trials or prompt rescue therapy with gilteritinib in the case of inadequate response; the combination of azacitidine + venetoclax + FLT3 inhibitor (not yet approved by any regulatory agency) should be considered when available”.

First round: 2 out of 8 (25%) panelists chose “Strongly agree”, 5 out of 8 (62.5%) chose “Agree”, and one panelist (12.5%) chose “Neither agree nor disagree”.

Patients with FLT3-mutated AML unfit for intensive chemotherapy have limited benefit with the combination of hypomethylating agents (HMAs) + Venetoclax (VEN) according to a genetic risk reclassification [36]. Early data demonstrated striking improvements with the addition of a FLT3i over historical doublet regimens. In a mixed prospective and retrospective cohort of 87 newly diagnosed FLT3-mutated older AML patients from MD Anderson, CR was 67% with triplet therapy vs. 32% with doublets (*p* = 0.002), and FLT3-PCR negativity 96% vs. 54% (*p* < 0.01) [37]. A single-center phase I/II study combining VEN + azacitidine (Aza) + gilteritinib in newly diagnosed patients (ND; *n* = 30) and in patients with relapsed/refractory (R/R; *n* = 22) FLT3-mutated (both TKD and ITD) AML reported cCR rates of 96% in ND patients [38], far exceeding the 59% reported in the intermediate-benefit group of the pooled analysis of phase Ib and phase III VEN-Aza trials [35,39,40]. Noteworthy, 65% of evaluable patients achieved deep molecular remission (FLT3-ITD MRD <1 × 10^−5^) within four cycles. Median OS had not been reached (18-month OS and OS rate is 71%) while in the VEN-Aza pooled analysis it was 12.1 months in the intermediate-benefit group. Although main grade ≥3 adverse events (infections in 53% of patients and febrile neutropenia in 33%) did not seem to differ with corresponding rates with doublet therapy (54% and 28%, respectively), prolonged myelosuppression was seen, which, however, resulted manageable with supportive strategies. A phase I/II trial investigated the optimal dosing and schedule of decitabine, venetoclax, and quizartinib triplet combination in 66 patients with FLT3-ITD mutated AML, of whom 19 newly diagnosed and 47 previously treated with FLT3i. The triplet demonstrated an activity in heavily pretreated and prior FLT3i-exposed (including 78% with prior gilteritinib exposure) patients, with a CRc rate of 68% and a median OS of 7.1 months. In the frontline setting, all patients achieved CRc without early deaths, median count recovery was 40 days, and median OS was not reached [41].

Ongoing phase III trials (comparing a sub-study of the NCI-sponsored myeloMATCH program: NCT06317649 and AMELIORATE trial: NCT04174612 sponsored by the Italian Group of hematological diseases in adults GIMEMA), and schedule-finding studies (SEQUENCE trial: NCT06696183 [42], VICEROY NCT05520567 [43]) will add more evidence to the existing data.

Notwithstanding the promising efficacy of the HMA + VEN + gilteritinib combination, the panelists considered that such treatment is not yet approved by any regulatory agency and that confirmation is needed in a multicenter and randomized setting to consolidate preliminary data. Given the availability of alternative options, such as enrollment in clinical trials, at this time triplet therapy should be considered within clinical trials. As data matures, this strategy may redefine frontline treatment for this genetically distinct AML subset.

### 3.2. FLT3 Inhibitors in First Line

Statement: “Intensive chemotherapy + midostaurin or quizartinib is the standard of care for patients with FLT3-ITD AML, eligible for intensive treatment.”

First round: 7 out of 8 (87.5%) panelists chose “Strongly agree” and one panelist (12.5%) chose “Disagree”.

Statement: “Intensive chemotherapy + midostaurin is the standard of care for patients with FLT3-TKD AML, eligible for intensive treatment.”

First round: 7 out of 8 (87.5%) panelists chose “Strongly agree” and one panelist (12.5%) chose “Agree”.

Evidence from pivotal trials supporting frontline use of midostaurin and quizartinib in combination with IC has been already presented [11,12]. Concerning the preference between midostaurin and quizartinib (given that both drugs are available), the panel emphasized that the two drugs have never been directly compared in head-to-head trials. However, several criteria can be recalled informing the choice between the two options. The QuANTUM-FIRST study included only patients with the FLT3-ITD mutation aged 18–75 years, while the RATIFY study included both patients with the FLT3-ITD and FLT3-TKD mutations but up to 60 years. The maintenance with quizartinib was provided to all patients with CRc for 36 months after the end of consolidation or allo-HSCT, while midostaurin could be continued for up to 1 year only in patients in CR1 who did not undergo allo-HSCT. The latter difference is mirrored in the regulatory label of the two drugs but midostaurin maintenance is not reimbursed in Italy, although it is allowed by the EMA label. Although the overall HR for OS relative to placebo was identical (0.78) in the two studies, subgroup analyses highlighted some notable differences. In patients with FLT3-ITD mutated AML, HR of midostaurin was 0.80–0.81 (for FLT3-ITD high vs. low allele burden), while quizartinib had better results in patients younger than 60 years (HR 0.68; 95% CI: 0.49–0.95); female patients seemed to benefit more from quizartinib (hazard ratio HR 0.69, 95% CI: 0.50–0.96), while midostaurin did not appear to confer any advantage in women (HR for OS: for males 0.54, 95% CI: 0.40–0.73 vs. 1.00, 95% CI: 0.75–1.33 for females). Also, the toxicity profile was different, with higher rate of myelosuppression and a more stringent threshold for QTc (<450 ms vs. <500 ms) with quizartinib, while midostaurin caused more severe nausea and cutaneous toxicity relative to placebo.

An open-label, multicenter phase IIIb trial was designed to further assess the safety and efficacy of midostaurin plus chemotherapy in induction, consolidation, and maintenance monotherapy in young (≤60 years) and older (>60 years) patients newly diagnosed with FLT3-mutated AML. Compared with RATIFY, this study prolonged midostaurin treatment from 14 days to 21 days, substituted anthracyclines (idarubicin or daunorubicin), and modified the standard combination chemotherapy dosing (“7 + 3” or “5 + 2” in more unfit patients). The CR + CRi rate was comparable between age groups (≤60 years 83.5%; >60 to ≤70 years 82.5%; in patients > 70 years 64.1%) and the type of anthracycline used in induction. Interestingly, in contrast to the RATIFY trial, the CR + CRi rate was lower in males (76.4%) than females (84.4%). No difference was seen in AE frequency between age groups, but grade ≥3AE frequency was higher in older patients [44].

After weighting the above-mentioned data with the caveat that cross-trial comparisons should be taken with caution, the panel deemed that quizartinib could be preferred, when available, for patients with FLT3-ITD, especially within the more favorable subgroups (women < 60 years), while midostaurin would remain the only available option for the group of FLT3-TKD-mutated patients.

The panel supported this recommendation based upon the more permissive label of quizartinib, allowing for maintenance irrespective of the use of allo-HSCT. As reminded earlier, trials are also ongoing to compare gilteritinib + intensive chemotherapy versus midostaurin + intensive chemotherapy [26,27] (Table 1).

### 3.3. MRD Monitoring and Impact on Therapeutic Decision-Making

Statement: “In patients with FLT3-mutated AML, MRD should be evaluated with available biomarkers: (i) qPCR to quantify NPM1 mutations is recommended when co-mutations in NPM1 gene are present; (ii) PCR-NGS to quantify FLT3-ITD should be performed, if available; (iii) multiparametric cytofluorimetry can be used in the absence of molecular biomarkers. Any positive finding is associated with a higher risk of relapse and should be managed accordingly”.

First round: 6 out of 8 (75%) panelists chose “Strongly agree” and two panelists chose “Agree”.

Currently, the two most extensively used methodologies to determine MRD are multiparameter flow cytometry-based MRD and molecular MRD. According to ELN guidelines [9], multiparameter flow cytometry-based MRD assessment should be performed with a qualified assay based on guidelines for rare event detection; this technique is applicable in more than 90% of AML cases with a sensitivity of 10^−4^–10^−5^. Molecular MRD should use a technique, including quantitative PCR (qPCR) and droplet PCR, that should reach a limit of detection of at least 10^−3^. Currently, qPCR can be used reliably only in AML with NPM1 mutations and in CBF AML, which account for approximately 30–45% of total cases. Leukemia-specific PCR assays (e.g., for NPM1, PML-RARA, or CBF AML) should be preferred over less specific markers, such as WT1 or EVI1. If using NGS, error-corrected targeted panel-based approaches should be preferred, and data should be interpreted in the context of variant-specific false-positive rates. Currently, NGS-based strategies are not standardized to be a stand-alone technique for MRD assessment [9,45].

FLT3-ITD MRD is a strong independent prognostic factor that identifies patients with AML at a high risk of relapse and death and overcomes the impact of other concomitantly established prognostic factors, including the NPM1 mutation and FLT3-ITD allelic ratio at diagnosis. During relapses, in most patients with AML in complete remission, FLT3-ITD MRD resulted as a clinically useful indicator for relapses, although FLT3-ITDs generally represent late events in AML development and can be lost or gained at relapse [46].

Retrospective studies using banked DNA samples for FLT3-ITD MRD analysis indicated that NGS-based approaches can reliably detect low levels of FLT3-ITD mutations in remission blood or bone marrow samples. These studies confirmed the prognostic utility of FLT3-ITD MRD in multiple clinical settings, including before allo-HSCT and described the presence of multiple FLT3-ITD clones (e.g., ITD mutations of different lengths in the same patient sample) in a substantial fraction of tested samples. The clinical significance of these multiclonal FLT3-ITD mutations has not been fully elucidated yet but studies are ongoing [47].

A dose-dependent relationship between persistent FLT3-ITD burden, before allo-HSCT, and post-transplant clinical outcomes was observed in patients with FLT3-ITD AML in first complete remission. Patients with the highest risk of relapse and death after transplant (approximately 1 in 6) can be recognized by a VAF MRD threshold of 0.01% or greater in pre-transplant remission blood using a commercially available testing kit for persistent FLT3-ITD (IVS; Invivoscribe, validated to detect a FLT3-ITD variant down to VAF of at least 0.005%). Patients with residual FLT3-ITD levels detected at lower VAFs had better outcomes, but still worse than those testing negative [48].

Beyond FLT3-specific MRD testing, any MRD-positive finding with available biomarkers should be considered as tied to a higher risk of relapse and should be managed accordingly. Indeed, the detection of molecular MRD through NGS and flow cytometry was associated with a significantly higher relapse rate than no detection (55.4% vs. 31.9%; HR, 2.14; *p* < 0.001), as well as with lower rates of relapse-free survival (36.6% vs. 58.1%; HR for relapse or death, 1.92; *p* < 0.001) and OS (41.9% vs. 66.1%; HR for death, 2.06; *p* < 0.001) [49]. In a systematic review and meta-analysis of MRD as a prognostic tool in AML among 11,151 patients treated intensively, the 5-year OS was 68% versus 34% among patients who achieved MRD negativity versus those who did not (average HR 0.36; 95% CI: 0.33–0.39) [50].

MRD-guided therapy in AML has been extensively explored in several studies, allowing for optimizing the therapeutic strategy and/or enabling pre-emptive treatments [51,52]. A thorough coverage of all possible uses of MRD testing in AML was beyond the scope of this work and this aspect was recently addressed in a recent policy review by the European Society for Blood and Marrow Transplantation Practice Harmonization and Guidelines Committee [53]. However, the panel emphasized that, although every MRD testing unavoidably provides some prognostic insight, its impact on the clinical decision making should not be absolute (i.e., black and white): as well as negative MRD results may not indicate complete disease eradication but refers to disease below the MRD test threshold in the tested sample, not all patients who are MRD positive will relapse. Therefore, although any MRD-positive finding is associated with a higher risk of relapse, this should be considered for clinical management in the context of a comprehensive evaluation of the patient and disease characteristics and its role in decision making should be personalized.

Statement: “Pre-transplant MRD evaluation is recommended since it helps define relapse risk and, therefore, it contributes to informing the decision about the most appropriate allogeneic transplantation platform for the individual patient”.

First round: 7 out of 8 (87.5%) panelists chose “Strongly agree” and one panelist (12.5%) chose “Agree”.

Several studies have explored the impact of pretransplant MRD combined with other variables on patients’ outcomes and were critically reviewed in a recent EBMT policy review providing detailed recommendations about this issue [53]. Generally, higher conditioning intensity has been proved beneficial in AML patients with genomic evidence of residual disease [54]. Specific data have confirmed this observation also within the context of FLT3-mutated AML. Grob et al. reported on 161 patients undergoing FLT3-ITD MRD evaluation with NGS; of these, 93 underwent allo-HSCT (30 with myeloablative conditioning -MAC- and 63 with reduced-intensity conditioning -RIC-) [46]. Although the overall risk of relapse was reduced in transplanted patients, an increased relapse incidence and worsen outcome was shown for those with positive FLT3-ITD MRD. In this group, MAC conditioning was associated with a reduced risk of relapse; given that NRM did not differ with conditioning intensity, the MAC group had improved OS in FLT3 MRD-positive patients.

A recent study analyzed data from the BMT CTN 1506 trial to evaluate the impact of transplant conditioning intensity, NPM1 mutations, and MRD on outcomes in FLT3-ITD-mutated AML [54]. Pretransplant MRD-negative patients showed no significant difference in relapse rates between MAC and RIC (HR: 1.202, 95% CI: 0.410–3.526). For MRD-positive, NPM1-wild-type patients, MAC appeared superior to RIC in reducing relapse risk (HR: 0.494, 95% CI: 0.240–1.016), though statistical significance was not formally reached. Patients with NPM1 mutations had the largest relapse-free survival (RFS) benefit from post-transplant gilteritinib (HR: 0.394, 95% CI: 0.181–0.855). In NPM1-mutated, MRD-positive patients, gilteritinib significantly reduced relapse risk (HR: 0.165, 95% CI: 0.048–0.564) [55].

Beyond conditioning intensity, other strategies of modulation of the allo-HSCT platform or proactive interventions have been explored to tackle the increased risk of relapse ensuing the finding of a positive peri-transplant MRD test, although not specifically designed for FLT3-mutated AML patients. The optimal donor selection strategy for such situation remains a matter of ongoing debate: as these patients may benefit from proceeding urgently to allo-HSCT, haplo-SCT may be a valuable choice, since evidence suggests that it is not detrimental but perhaps beneficial in abrogating the negative effect of positive pre-transplant MRD [56,57]. Early tapering of immunosuppression is known to hasten the Graft-versus-Leukemia effect, a key mechanism in preventing relapses, although at the cost of higher risk of developing or worsening an ongoing Graft-versus-Host Disease [58,59]. Furthermore, strict monitoring for molecular markers allows for a preemptive approach aimed at reversing molecular relapses before overt hematologic recurrence, with treatments like Donor Lymphocyte Infusion [60,61] which can be combined with FLT3i [62].

In conclusion, MRD status interacts with several parameters which can be modulated within the allo-HSCT platform (e.g., donor selection, conditioning intensity, immunosuppression tapering, post-HSCT management, etc.), and thus it should be considered to tailor the more appropriate choices to the individual patient.

### 3.4. Role of FLT3 Inhibitors in Relapsed and Refractory Patients

Statement: “Retesting of FLT3 mutational status is recommended in refractory-relapsed AML, regardless of the FLT3 mutational status at diagnosis”.

First round: 8 out of 8 (100%) panelists chose “Strongly agree”.

Statement: “Gilteritinib represents the treatment of choice in patients with refractory-relapsed FLT3-mutated AML, regardless of previous use of other FLT3i”.

First round: 7 out of 8 (87.5%) panelists chose “Strongly agree” and one panelist (12.5%) chose “Neither agree nor disagree”.

Statement: “For patients with R/R FLT3-mutated AML achieving response to gilteritinib, allogeneic HSCT must be evaluated as a potentially curative intervention”.

First round: 7 out of 8 (87.5%) panelists chose “Strongly agree” and one panelist (12.5%) chose “Agree”.

Statement: “For patients with R/R FLT3-mutated AML, gilteritinib should be considered as a bridge-to-transplant therapy and for post-transplant maintenance, given its clear therapeutic benefit in this setting of patients”.

First round: 4 out of 8 (50%) panelists chose “Strongly agree”, 3 out of 8 (37.5%) chose “Agree”, and one panelist chose “Neither agree nor disagree”.

Statement: “Gilteritinib is an effective option to treat FLT3-mutated AML relapsed after allogeneic transplant and should be considered early in this setting”.

First round: 6 out of 8 (75%) panelists chose “Strongly agree”, one panelist chose (12.5%) “Agree”, and another (12.5%) chose “Neither agree nor disagree”.

Gilteritinib is the only approved FLT3i in patients with refractory-relapsed FLT3-mutated AML, based on the results of the ADMIRAL trial [63]. The trial randomized 247 patients to gilteritinib and 124 to salvage chemotherapy and demonstrated the superior efficacy of gilteritinib monotherapy. This treatment achieved a cCR rate of 34.0% vs. 15.3% with chemotherapy, with 25.5% of responders successfully bridging to allo-HSCT (vs. 15.3% in the standard arm). Median OS was significantly longer with gilteritinib than with chemotherapy (9.3 vs. 5.6 months; HR 0.64; 95% CI: 0.49–0.83; *p* < 0.001). Gilteritinib real-world results mirrored those of clinical trials. In a UK cohort (*n* = 152), median OS was 9.5 months, with CR achieved in 21% of the cohort that included patients with at least 2 prior lines of therapy (36%), such as FLT3i (41%) and venetoclax (24%) [64]. An Italian retrospective study of 205 patients with relapsed (*n* = 148) or refractory (*n* = 57) FLT3-mutated AML (both TKD and ITD) treated with gilteritinib monotherapy across 27 centers reported a CR rate of 43.4% and CRi of 8.8%, with a median OS of 10.3 months. Among patients receiving gilteritinib as a bridge to allo-HSCT, CR and CRi rates were 52.4% and 10.5%, respectively, with a median OS of 11 months. Overall, 48% of transplant-eligible patients effectively underwent transplantation after a median of 3.7 months, with post-transplant survival rates of 65.2% at 1 year and 56.1% at 3 years [65].

In FLT3-mutated AML, a potential concern is that prior use of other FLT3i may determine the development of resistance. Gilteritinib inhibits FLT3 by binding the ATP-binding site, and its activity is not affected by mutations occurring in the activation loop of TKD, such as D835, which may arise during FLT3 inhibition with type II inhibitors, sorafenib and quizartinib. Theoretically, gilteritinib (as well as all other FLT3i) may be significantly less effective in the presence of mutations at the gatekeeper residue F691 or deregulation/hyperactivation of parallel and downstream pathways, such as RAS [66].

A comprehensive assessment of pattern of clonal evolution after front-line therapy with a FLT3i was first reported by Schmalbrock et al. [67]: 54 paired samples from diagnosis and at time of relapsed-refractory disease were obtained from patients treated with midostaurin + IC in first line from RATIFY and AMLSG16-10 trials. In the second sample, 46% of patients became FLT3-ITD negative but acquired mutations in signaling pathways (e.g., MAPK), thereby providing a new proliferative advantage. In cases with FLT3-ITD persistence, the selection of resistant ITD clones was found in 11% as potential drivers of disease. In 32% of cases, no FLT3-ITD mutational change was observed, suggesting either resistance mechanisms bypassing FLT3 inhibition or loss of midostaurin inhibitory activity because of inadequate drug levels. More recently, Arora et al. [68] evaluated mechanisms of relapse in 272 patients undergoing first line treatment with IC + FLT3i (*n* = 107) or lower intensity treatment (LIT, *n* = 165; of which 93 with HMA + VEN + FLT3i), of which 215 had FLT3-ITD mutation, 31 had FLT3-TKD and 26 had both FLT3-ITD and TKD mutations. Eighty patients relapsed: a loss of FLT3-ITD mutations was noted in 27/67 (40%) evaluable patients, a loss of FLT3-TKD in 8/13 (62%); a total of 28/72 patients (39%) tested negative for both FLT3-ITD and FLT3-TKD (FLT3-wild-type relapses). Use of different FLT3i, consolidation with allo-HSCT or maintenance with a FLT3i did not impact the rate of FLT3-wild-type relapses. However, there was a trend toward higher rates of FLT3-wild-type relapses in transplanted patients who received post-transplant FLT3i maintenance compared to those who did not (58% versus 20%, *p*  =  0.10). Among relapsed patients who were wild-type FLT3-TKD at baseline, 8/59 (14%) had a new emergent FLT3-TKD mutation (median VAF of 29%); 88% of them had received frontline type II FLT3i, which are not active against FLT3-TKD mutations (sorafenib in 5, quizartinib in 2) [68]. Emergent non-FLT3 mutations were detected in 28/56 (50%) evaluable patients, the most common being DNA methylation mutations (DNMT3A, TET2, IDH1, IDH2) in 21%, RAS mutations in 16%. Accordingly, FLT3 mutations may arise or become detectable at the time of relapse in patients who were negative or positive at low levels at initial diagnosis, in the context of clonal evolution [65,69]. These data indicate the need to re-test FLT3 mutations at relapse or in the case of refractory disease, independent of the FLT3 mutational status at diagnosis.

However, accumulating evidence supports the effectiveness of gilteritinib even after previous use of other FLT3i. In the ADMIRAL trial, superiority of gilteritinib monotherapy over salvage chemotherapy was maintained, although narrowed, in those who had previously received an upfront FLT3 inhibitor [70]. Data collected in the context of the Italian Expanded Access Program for gilteritinib showed that, among patients who had previously received midostaurin or sorafenib, the median OS was 13.2 months, with 1-year and 3-year survival rates of 51.3% (95% CI, 42.4–62.2) and 33.9% (95% CI, 25.4–45.1), respectively. In contrast, in patients without prior TKI exposure, median OS was 8.0 months, with 1-year and 3-year survival rates of 38.4% (95% CI, 26.9–54.9) and 11.1% (95% CI, 4.6–27.8), respectively. These data may not be conclusive because of the presence of some confounding factors, such as the younger age and the lower ELN 2017 risk of patients previously treated with TKI; however, prior treatment with a FLT3i seems to be associated with significantly improved survival compared with patients without such exposure [65].

A French ambispective study evaluated the real-world effectiveness and safety of single-agent gilteritinib in 167 R/R FLT3-mutated AML patients, including 67 previously treated by intensive chemotherapy and midostaurin. The rates of cCR and median OS did not differ upon previous use of midostaurin (in this group cCR was 27.5% and median OS 7.8 months) and were comparable to those seen in the ADMIRAL trial. Multivariate analysis indicated a reduction in mortality risk by 87% with allogenic HSCT after gilteritinib (HR 0.13) [71].

With respect to post-transplant use of FLT3i, the randomized, double-blinded, placebo-controlled Blood and Marrow Transplant Clinical Trials Network (BMT CTN) 1506 (MORPHO) trial specifically investigated the role of gilteritinib in post-transplant maintenance in patients with FLT3-ITD mutated AML in first remission with not more than two cycles of intensive therapy (with or without a FLT3i) [47]. In the primary analysis of the MORPHO trial, patients with peri-transplantation positive MRD (i.e., detectable MRD either immediately before, or immediately after HSCT and before randomization using a 1 × 10^−6^) had a reduced rate of relapse if they were on the gilteritinib arm (HR for RFS was 0.515 in favor of the gilteritinib arm; *p* = 0.0065). Based on this result, gilteritinib may be re-initiated in patients following allo-HSCT, and the treatment should continue until the patient has a clinical benefit or until unacceptable toxicity occurs. Moreover, the importance of regular monitoring of patients for MRD should be reinforced for FLT3-ITD MRD undergoing allo-HSCT [72].

In patients with R/R-AML treated with gilteritinib as bridge-to-transplant therapy, gilteritinib provided some benefits when reintroduced post-transplant and, therefore, could be potentially considered for such use.

The ADMIRAL trial suggested that reinduction with gilteritinib after allo-HSCT may be a feasible option for fit patients. In the study, 64 patients (out of 247 randomized to gilteritinib arm) could undergo transplantation; of them, 40 resumed gilteritinib after transplantation, and 16 were still alive without relapse after at least 2 years from randomization, in comparison to 2 of 24 who did not resume gilteritinib after transplantation (median OS for the two groups was 16.2 vs. 8.4 months) [12,48].

### 3.5. Toxicity Management and Therapy Optimization

Statement: “Gilteritinib dosing should follow instructions reported in the Summary of Product Characteristics; however, if a dose-limiting toxicity occurs it is advisable to lower the dose rather than discontinue the drug”.

First round: 7 out of 8 (87.5%) panelists chose “Strongly agree” and one panelist (12.5%) chose “Agree”.

For optimal therapeutic efficacy, FLT3 inhibition should be near-complete and long-lasting. This is pharmacokinetically supported by the drug’s long elimination half-life of approximately 113 h; therefore, allowing for once-daily dosing. Given this requirement for sustained target engagement, it is reasonable to minimize drug interruptions by favoring dose reduction over discontinuation. For the same reason, as reported in the SmPC, if CR is not reached after 4 weeks of treatment, dosing should be increased to 200 mg daily (Table 2).

## 4. Discussion

The landscape of FLT3-mutated AML has undergone a paradigm shift following the introduction and successful integration of FLT3i into standard care. The demonstrated improvement in patient outcomes, particularly in the frontline setting, has repositioned FLT3-mutated AML into a more favorable intermediate prognostic category (per the ELN classification), enhancing eligibility for curative strategies such as allo-HSCT. Furthermore, the availability of highly selective FLT3i has provided a significant therapeutic breakthrough for patients with relapsed/refractory disease.

Such rapid innovations inevitably lead to an increase in clinical practice heterogeneity, as centers must constantly adapt to emerging data. The need for a formal consensus arises precisely from this observed variability; even when experts agree on broad principles, their standardized application across different institutions is often lacking. Consequently, we addressed the urgent need for a structured clinical guide in the face of this rapid therapeutic evolution, providing pragmatic, immediately implementable recommendations for the clinical context. In this setting, while the ELN 2022 guidelines offer a general framework, they primarily provide broad guidance with directives interspersed throughout the narrative text. Our work, therefore, serves as a methodological expansion specifically tailored to the FLT3-mutated landscape, translating evidence into standalone “actionable” statements. Although narrative reviews abound on this topic, the value of these recommendations lies not in the novelty of presented data, but in their clinical utility—specifically the valid translation of established evidence into specific clinical actions through a structured, comprehensive set of practice-oriented recommendations derived through a rigorous modified Delphi approach.

Our panelists, representing leading Italian experts, achieved a high threshold of agreement in the first round. Rather than implying a lack of debate, this degree of concordance, likely reflects the rigorous preliminary work performed by the steering committee in identifying “hot topics” and drafting precise, evidence-based statements. Despite the high agreement, the panel addressed several nuanced and debated topics, including the specific role of allo-HSCT in FLT3-ITD patients, optimal strategies for “unfit” patients, and the evolving use of gilteritinib as post-transplant maintenance. The whole process resulted in a consensus aligned with major international bodies, thereby reinforcing the reliability of these recommendations for the hematological community. Although a formal systematic review was not performed, a leaner methodological approach allowed for a comprehensive coverage of the entire clinical continuum, ranging from initial diagnostic testing and prognostic classification to the precise scheduling of upfront FLT3i integration, indication, timing and clinical choices for allo-HSCT, clinical use of MRD and finally, the intricate decisions regarding FLT3i maintenance and R/R strategies. In a field defined by rapid data generation, this structured consensus benefits current knowledge by translating complex trial data and biological understanding into clear, actionable clinical recommendations which can be immediately implemented in clinical settings, ultimately optimizing patient outcomes in this high-risk population.

Looking forward, the next therapeutic frontier lies in consolidating promising data about triplet combinations and simultaneous targeting of co-mutational pathways. As an example, the high co-occurrence of FLT3-mutations with key primary drivers like NPM1 mutations and KMT2A (MLL) rearrangements highlights the rationale for combining FLT3i (e.g., gilteritinib or quizartinib) with Menin inhibitors [73,74]. This dual-targeting strategy promises to synergistically dampen proliferative signals (driven by FLT3) while releasing the differentiation block (driven by Menin/KMT2A), a hypothesis currently being explored in clinical trials with promising initial efficacy [75,76]. Concurrently, novel immunotherapeutic approaches like anti-FLT3 CAR-T cells and bispecific antibodies are under active development, offering further options for patients with RR-AML [77,78,79]. Ultimately, the future of FLT3-mutated AML management is defined by further optimization of current therapeutic toolkit and precision and combination treatments, leveraging initial clinical success to pursue deeper, more durable remissions through multimodal and targeted approaches.

## 5. Conclusions

The integration of FLT3i has shifted FLT3m-AML into a more favorable intermediate prognostic category, enhancing the role of curative strategies like allo-HSCT. This consensus paper provides a structured evidence-based comprehensive guide, translating complex data into clear actionable clinical recommendations that minimize practice variability and ultimately optimize management for this high-risk population.

## Figures and Tables

**Table 1:** Randomized trials in patients with FLT3-mutated AML, newly diagnosed (completed or ongoing).

Trial	Design	Study Population	Treatment	Primary Endpoint	Results
CALGB 10603/RATIFY NCT00651261 QUANTUM First	Randomized, placebo-controlled, double blind, multicenter, phase III trial	Fit patients with FLT3-mutated AML (ITD or TKD), 18–59 years *n* = 360 in the midostaurin group (M) *n* = 357 in the placebo group (P)	IC (7 + 3 induction + HDAC consolidation) + either M or P followed by 1-year maintenance (M or P) if remission (allo-HSCT excluded)	Reached: HR for OS 0.78 (95% CI: 0.63 to 0.96; *p* = 0.009)	mOS (months): 74.7 (M) vs. 25.6 (P)
NCT02668653	Randomized, double-blind, placebo-controlled, phase III trial	Fit patients with FLT3-ITD-mutated AML *n* = 268 in the quizartinib group (Q) *n* = 271 in the placebo group (P)	IC (7 + 3 induction + HDAC consolidation +/− allo-HSCT) + either Q or P followed by 3-year continuation (Q or P)	Reached: HR for OS 0.78 (95% CI: 0.62 to 0.98; *p* = 0.032)	mEFS (months): 26.7 (M) vs. 15.5 (P)
CALGB 10603/RATIFY	Randomized, open-label phase II study	Fit patients with FLT3-mutated AML (ITD or TKD), 18–70 years *n* = 90 in the gilteritinib group (G) *n* = 87 in the midostaurin group (M)	IC (7 + 3 induction + HDAC consolidation) + either G or M (maintenance not included)	Not reached: post-induction FLT3m-MRD negative: 64.4% (G) vs. 59.8% (M), *p* = 0.539	CR: 58.9% (M) vs. 53.5 (P)
HOVON-156/AMLSG 28-18 PASHA NCT04027309	Randomized, open-label phase III study	Newly diagnosed, fit FLT3-ITD or FLT3-TKD (D835/I836), aged > 18 y *n* = 777	Gilteritinib + IC versus midostaurin + IC 7 + 3 plus TKI induction for up to 2 cycles followed by age adapted	EFS	Ongoing, not recruiting
MM1OA-EA02	Randomized, open-label phase II study	Newly diagnosed, FLT3-ITD or FLT3-TKD (D835/I836), aged > 60 y, or unfit *n* = 149	Consolidation plus TKI, followed by maintenance	OS	Recruiting
NCT06317649	Randomized, open-label phase II study	Newly diagnosed, FLT3-ITD or FLT3-TKD (D835/I836) aged >60 y *n* = 208	Gilteritinib + venetoclax + azacitidine versus Venetoclax + azacitidine	CR without MRD rate	Ongoing

mEFS, modified event-free survival; mOS, modified overall survival; MRD, minimal residual disease; CR, complete response; TKI, tyrosine kinase inhibitor; ITD, internal tandem duplication; TKD, tyrosine kinase domain; AML, acute myeloid leukemia; HSCT, hematopoietic stem cell transplantation.

**Table 2:** Comparison between midostaurin, quizartinib, and gilteritinib.

	Midostaurin	Quizartinib	Gilteritinib
Indication (mutation)	FLT3-ITD/FLT3-TKD	FLT3-ITD	FLT3-ITD/FLT3-TKD
Indication (setting)	First line with IC	First line with IC	R/R in monotherapy until PD
Maintenance after chemotherapy	Approved	Approved	N/A
Maintenance after allo-HSCT	Not approved	Approved	Approved
QTc(F)	<500 ms	<450 ms	<500 ms
Age	Data available 18–70 years	Data available 18–75 years	Data available 20–84 years
HR (95% CI) in patients >60 years vs. standard arm	0.42 (0.29–0.61) °	0.91 (0.66–1.26)	0.64 (0.44–0.95) *
Favored subgroups (OS) in pivotal trials	Males	Females	Females

° Comparison to historical controls. * Patients >65 years.

## Data Availability

The original contributions presented in this study are included in the article. Further inquiries can be directed to the corresponding author.

## Abbreviations

The following abbreviations are used in this manuscript:

AML	Acute Myeloid Leukemia
CR	Complete Response
CRc	Composite Complete Response
DLT	Dose-Limiting Toxicity
EFS	Event Free Survival
HSCT	Hematopoietic Stem Cell Transplant
OS	Overall Survival
RFS	Relapse Free Survival

## Appendix A. Search Strategy

Step 1: Define Core Concepts

1. Trials or Studies

MeSH terms:

Clinical Trial[Publication Type]

Clinical Trials as Topic[MeSH]

Randomized Controlled Trial[Publication Type]

Free-text terms:

trial*

study OR studies

random*

phase 1 OR phase 2 OR phase 3

2. FLT3 Mutation

MeSH terms:

fms-Like Tyrosine Kinase 3[MeSH] (covers FLT3 gene)

Mutation[MeSH]

Free-text terms (spelling variants):

FLT3 OR FLT-3

FLT3 mutat* OR FLT3-ITD OR FLT3-TKD

“FMS-like tyrosine kinase 3”

3. Acute Myeloid Leukemia (AML)

MeSH terms:

Leukemia, Myeloid, Acute[MeSH]

Free-text terms:

AML OR “acute myeloid leuk*” OR “acute myelogenous leuk*”

4. Frontline or Relapsed/Refractory Setting

Frontline terms:

first-line OR frontline OR “previously untreated” OR induction

newly diagnosed

Relapsed/Refractory terms:

relaps* OR refractory OR recurrent

salvage OR “advanced disease”

Step 2: Combine Concepts with Boolean Operators

(Clinical Trial[Publication Type] OR Clinical Trials as Topic[MeSH] OR Randomized Controlled Trial[Publication Type] OR trial* OR study OR studies OR random* OR “phase 1” OR “phase 2” OR “phase 3”)

AND

(fms-Like Tyrosine Kinase 3[MeSH] OR Mutation[MeSH] OR FLT3 OR “FLT-3” OR “FLT3 mutat*” OR FLT3-ITD OR FLT3-TKD OR “FMS-like tyrosine kinase 3”)

AND

(Leukemia, Myeloid, Acute[MeSH] OR AML OR “acute myeloid leuk*” OR “acute myelogenous leuk*”)

AND

(first-line OR frontline OR “previously untreated” OR induction OR “newly diagnosed”) OR (relaps* OR refractory OR recurrent OR salvage OR “advanced disease”)

AND

“FLT3 inhibitor*” OR Midostaurin OR gilteritinib OR Quizartinib OR Sorafenib OR crenolanib OR “tyrosine kinase inhibitor*” OR TKI)

Filters:

Limit to human studies and English language.

Publication date: limit last 10 years.

## Appendix B. List of Final Statements

1. Diagnosis, risk stratification, and first therapeutic decision

1.1 FLT3-ITD mutation analysis is recommended at diagnosis in all AML patients eligible for antileukemic treatment. Capillary electrophoresis is the detection method of choice. Next Generation Sequencing (NGS) is an alternative option, being equally informative when positive.

1.2 FLT3-TKD mutation analysis is recommended at diagnosis in all AML patients eligible for antileukemic treatment. Capillary electrophoresis is the detection method of choice. NGS is an alternative option, being equally informative when positive.

1.3 All patients with FLT3-mutated AML who are fit for intensive chemotherapy must receive intensive chemotherapy plus a FLT3i regardless of other co-occurring mutations.

1.4 All patients with FLT3-ITD AML who are fit for intensive chemotherapy should be evaluated for allogeneic transplant eligibility.

1.5 Current standard treatment for patients with FLT3-ITD AML who are unfit for intensive chemotherapy is represented by the combination of a hypomethylating agent and venetoclax, which, however, should be considered suboptimal in this subset of AML. Alternative options include enrollment in clinical trials or prompt rescue therapy with gilteritinib in the case of inadequate response; the combination of azacitidine + venetoclax + FLT3 inhibitor (not yet approved by any regulatory agency) should be considered when available.

2. FLT3 inhibitors in first line

2.1 Intensive chemotherapy + midostaurin or quizartinib is the standard of care for patients with FLT3-ITD AML, eligible for intensive treatment.

2.2 Intensive chemotherapy + midostaurin is the standard of care for patients with FLT3-TKD AML, eligible for intensive treatment.

3. MRD monitoring and impact on therapeutic decision-making

3.1 In patients with FLT3-mutated AML, MRD should be evaluated with available biomarkers: (i) qPCR to quantify NPM1 mutations is recommended when co-mutations in NPM1 gene are present; (ii) PCR-NGS to quantify FLT3-ITD should be performed, if available; (iii) multiparametric cytofluorimetry can be used in the absence of molecular biomarkers. Any positive finding is associated with a higher risk of relapse and should be managed accordingly.

3.2 Pre-transplant MRD evaluation is recommended since it helps in defining relapse risk and, therefore, it contributes to inform the decision about the most appropriate allogeneic transplantation platform for the individual patient.

4. Role of FLT3 inhibitors in relapsed and refractory patients

4.1 Retesting of FLT3 mutational status is recommended in refractory-relapsed AML, regardless of the FLT3 mutational status at diagnosis.

4.2 Gilteritinib represents the treatment of choice in patients with refractory-relapsed FLT3-mutated AML, regardless of previous use of other FLT3i.

4.3 For patients with R/R FLT3-mutated AML achieving response to gilteritinib, allogeneic HSCT must be evaluated as a potentially curative intervention.

4.4 For patients with R/R FLT3-mutated AML, gilteritinib should be considered as a bridge-to-transplant therapy and for post-transplant maintenance, given its clear therapeutic benefit in this setting of patients.

4.5 Gilteritinib is an effective option to treat FLT3-mutated AML relapsed after allogeneic transplant and should be considered early in this setting.

5. Toxicity management and therapy optimization

5.1 Gilteritinib dosing should follow instructions reported in the Summary of Product Characteristics; however, if a dose-limiting toxicity occurs it is advisable to lower the dose rather than discontinue the drug.

## Appendix C. List of Statements Initially Voted by the Panelists

The original statements proposed by the steering committee and voted by the panelists are reported below. A brief summary of subsequent changes and refinements discussed by the panelists, leading to final wording, is reported in brackets.

1. Diagnosis, risk stratification, and first therapeutic decision

1.1a FLT3-ITD mutation analysis (preferentially by capillary electrophoresis) is recommended at diagnosis in all AML patients eligible for antileukemic treatment.

1.1b FLT3-TKD analysis (preferentially by capillary electrophoresis) is suggested at diagnosis in all AML patients eligible for antileukemic treatment.

(The panel decided to clarify the different role of the two techniques in the final version)

1.2 ELN recommendations 2022 are the appropriate framework for risk stratification in patients with FLT3-mutated AML. Regardless of the FLT3 allelic ratio (area under the curve of FLT3-ITD/area under the curve of FLT3-wild-type) or the presence of an NPM1 co-mutation, these guidelines link FLT3 mutations to an intermediate risk.

(This statement was deleted as it was deemed obvious)

1.3 All patients with FLT3-ITD AML who are fit for intensive chemotherapy must receive intensive chemotherapy plus an FLT3 inhibitor and should be considered candidates for allogeneic transplant.

(This statement was split for enhanced clarity)

1.4 Current standard treatment for patients with FLT3-ITD AML who are unfit for intensive chemotherapy is represented by the combination of a hypomethylating agent and venetoclax; as this treatment cannot be considered optimal in FLT3-ITD AML, the combination of azacitidine + venetoclax + FLT3 inhibitor should be considered in patients with FLT3-ITD AML unfit for intensive chemotherapy.

(Substantial refinements were made to this statement to clarify the regulatory status of the triplet combination and to consider all the available alternative options)

2. FLT3 inhibitors in first line

2.1 Intensive chemotherapy + midostaurin is the standard of care for patients with FLT3-mutated AML, eligible for intensive treatment.

2.2 Intensive chemotherapy + quizartinib is a forthcoming option for patients with FLT3-ITD AML, eligible for intensive treatment.

(These statements were rephrased to focus on the patient population, i.e., the type of FLT3 mutation, instead of the treatment regimen)

3. MRD monitoring and impact on therapeutic decision-making

3.1 In patients with FLT3-mutated AML, MRD assessment should be performed using available markers: if the PCR-NGS method for FLT3-ITD is available, no other markers are required; if a concomitant NPM1 mutation is present, it may be performed via NPM1 PCR; if no other markers are available, multiparametric flow cytometry should be used.

(The hierarchy of options has been modified to favor those currently widely available, and a note on the general interpretation of MRD—detailed in the rationale for the recommendation—has been included)

3.2 Pre-transplant MRD evaluation is recommended since it helps to define relapse risk and, therefore, it contributes to plan the most appropriate allogenic transplantation platform for the individual patient.

(Minor refinements)

4. Role of FLT3 inhibitors in relapsed and refractory patients

4.1 Gilteritinib represents the treatment of choice in patients with refractory-relapsed FLT3-mutated AML, regardless of the use of midostaurin in the first line.

(No change)

4.2 Further testing of FLT3 mutational status is recommended in refractory-relapsed AML, regardless of the FLT3 mutational status at diagnosis.

(No change)

4.3 For patients with R/R FLT3-mutated AML achieving response to gilteritinib, allogeneic HSCT should be evaluated as a potentially curative intervention.

(No change)

4.4 In patients with relapsed/refractory AML who received gilteritinib as a bridge-to-transplant therapy, the drug showed a benefit in the post-transplant recovery and, consequently, should be considered for such use.

(The statement was rephrased to make it more actionable)

4.5 Gilteritinib is an effective option to treat FLT3-mutated AML relapsing after allogeneic transplant and should be considered early in this setting.

(No change)

5. Toxicity management and therapy optimization

5.1 Gilteritinib dosing should follow instructions reported in the Summary of Product Characteristics; however, if a dose-limiting toxicity occurs it is advisable to lower the dose rather than discontinue the drug. Dose re-escalation should be considered once the toxicity has resolved.

(The last sentence was removed as it was deemed obvious)
